# Psychosocial and structural factors associated with postpartum depressive symptoms and maternal continuum of care in Borama, Somaliland: a cross-sectional study

**DOI:** 10.1080/16549716.2026.2741766

**Published:** 2026-10-07

**Authors:** Abdikerim Mohamed Moumin, Abdalle Ali Yusuf, Abdirizak Mohamed Moumin, Khadar Mowlid Abdi, Bishar Elmi Yey, Hibak Ahmed Nuh, Abdifatah Ahmed Osman

**Affiliations:** a School of Postgraduate, Amoud University, Borama, Somaliland; b Faculty of Business and Economics, Amoud University, Borama, Somaliland; c School of Public Health and Nutrition, Amoud University, Borama, Somaliland

**Keywords:** Perinatal mental health, spousal support, postnatal care, health service utilization, Social Support Theory

## Abstract

**Background:**

Postpartum depression (PPD) and fragmented maternal care are critical public health challenges in sub-Saharan Africa.

**Objective:**

To examine the associations of psychosocial, structural, clinical, and health-system factors with self-reported postpartum depressive symptoms and maternal continuum-of-care (CoC) completion in Borama, Somaliland.

**Methods:**

A community-based cross-sectional study analyzed 442 postnatal women. Postpartum depressive symptoms were assessed via binary self-report, and CoC was summarized using a 0–3 index (ANC4+, facility delivery, and postnatal care). Multivariable logistic regression estimated adjusted odds ratios (aORs) for depressive symptoms; robust multiple linear regression estimated adjusted CoC-score differences (N=442).

**Results:**

Depressive symptoms were reported by 109/442 women (24.7%), and 152/442 (34.4%) completed all three CoC components. Higher adjusted odds of symptoms were observed among single mothers (aOR = 2.27, 95% CI 1.11–4.65), women with labor complications (aOR = 2.06, 95% CI 1.10–3.87), university/technical education (aOR = 2.49, 95% CI 1.16–5.33), and fair/poor self-rated health (aOR = 6.66, 95% CI 2.62–16.90). Among symptom-positive women, 37.6% spoke to no one about their distress. Adjusted symptom probabilities declined across increasing spousal-support categories, but the overall association was not statistically significant (p=.430). Early ANC initiation (B=0.345, 95% CI 0.205–0.486) and university/technical education (B=0.382, 95% CI 0.182–0.582) were positively associated with the CoC score.

**Conclusions:**

Postpartum depressive symptoms and incomplete continuity of care were prevalent in this sample. Maternal-health planning should consider integrating routine emotional symptom enquiry with strategies that strengthen early ANC engagement and postnatal follow-up.

## Paper Context

- **Main findings**: In this cross-sectional study of postnatal mothers in Borama, Somaliland, nearly one-quarter reported postpartum depressive symptoms, while only about one-third completed all three stages of the maternal continuum of care.
- **Added knowledge**: Early initiation of antenatal care showed the strongest positive association with completing the full maternal care continuum, while self-rated poor health, labor complications, single motherhood, and higher educational attainment were associated with higher odds of postpartum depressive symptoms.
- **Global health impact for policy and action**: Maternal health programs in post-conflict and resource-constrained settings should integrate routine mental health symptom enquiry into maternal services and strengthen early entry into antenatal care to support continuity across the postpartum period.

## Background

Postpartum depression (PPD) is a critical global public health issue affecting approximately 17.22% of women globally, with significant regional variations, such as the 39.96% prevalence observed in Southern Africa [1]. In low- and middle-income countries (LMICs), the burden is particularly heavy, as approximately one in four women (24.7%) suffer from perinatal depression [2]. Within sub-Saharan Africa (SSA), the pooled prevalence of PPD symptoms is estimated at 22.1%, while maternal mental-health needs remain insufficiently integrated into routine maternal services in many settings, particularly where screening, referral pathways, and specialist capacity are limited [3,4]. Regional estimates across East Africa demonstrate substantial variation depending on setting, screening tools, and cutoffs; for example, prevalence ranges from approximately 15.6% to 24.0% in Ethiopia and up to 27.1% in southwestern Uganda, with estimates varying significantly whether using the Edinburgh Postnatal Depression Scale (EPDS) at a cutoff of ≥10 or ≥13 [5–7]. Improving the detection and management of maternal mental health is vital for achieving Sustainable Development Goal 3 (SDG 3), which targets the reduction of maternal mortality and promotion of mental wellbeing [8].

Maternal continuum of care (CoC) is a strategic framework that integrates health services from pregnancy through childbirth to the postnatal period [9]. This pathway includes at least four antenatal care visits (ANC4+), skilled birth attendance (SBA), and postnatal care (PNC) within 42 days of delivery [10]. Despite the clinical advantages of continuous care, completion rates in SSA are remarkably low, with only 18.72% of women utilizing the full continuum in some meta-analyses [11]. Multi-country evidence across 17 sub-Saharan African nations indicates that structural bottlenecks – including distance to facilities, low household wealth, limited maternal autonomy, lack of health insurance, and suboptimal or disrespectful provider interactions – drive widespread attrition along the continuum [12]. Significant wealth-related inequalities occur during the transition to the postpartum stage, which is hazardous because over 65% of maternal deaths occur during this window [13]. Research indicates that irregular service utilization and dropouts from the continuum are significantly associated with increased odds of developing PPD [14,15]. Conversely, consistent engagement with the care continuum facilitates early screening and provides essential psychological support [16,17]. However, the relationship between clinical care pathways and mental health remains underexplored, necessitating a dual analysis that treats health system utilization and psychological well-being as interdependent outcomes.

Examining these outcomes together within the same population is therefore important because continuity of maternal care provides repeated opportunities for contact with health services, while psychosocial distress may coexist with barriers to sustained care engagement; in this cross-sectional study, these relationships are interpreted as associations rather than as evidence of temporal or causal pathways [14–17]. A combined analysis is particularly necessary in resource-limited African contexts where service fragmentation deprives mothers of routine mental health screening, while untreated postpartum depressive distress can diminish maternal agency and motivation to sustain recommended postnatal visits, creating a bidirectional cycle of disengagement.

Social support serves as a multidimensional protective mechanism that can buffer the onset of PPD symptoms [18]. Grounded in Social Support Theory [19], this construct encompasses emotional, instrumental, and informational assistance from social networks. In SSA, inadequate social support is a leading correlate of PPD [20]. Women who report poor partner support are significantly more likely to experience PPD [21], while those lacking support from family or friends face significantly higher risks [7,22]. Recent empirical investigations from West Africa further demonstrate that key overlapping determinants – such as poor partner support, physical delivery complications, intimate partner disharmony, domestic abuse, food insecurity, and household poverty – substantially heighten maternal vulnerability to postpartum distress [23,24]. In countries, such as Ethiopia and Uganda, perceived support from relatives and high-quality friend networks has been shown to facilitate maternal resilience and improve health-seeking behaviors [5]. In this context, spousal support and household assistance are critical for preventing severe mental health outcomes and ensuring clinical adherence.

Postpartum depression assessment in sub-Saharan Africa primarily utilizes scales such as the Edinburgh Postnatal Depression Scale (EPDS) and the Patient Health Questionnaire-9 (PHQ-9) [6]. Although the DSM-5 provides the definitive gold standard for clinical diagnosis, its application is often limited to resource-constrained settings, making validated self-report symptom assessments essential for identifying at-risk mothers. Accordingly, the outcome in this study is described in the Introduction as self-reported postpartum depressive symptoms rather than as a clinician-confirmed diagnosis; detailed measurement and coding procedures are reported in the Materials and Methods section.

The relationships examined in this study are conceptually grounded in the Stress Process Model [25], which provides a framework for considering how life stressors such as financial worry, labor complications, and single motherhood may operate alongside available social and clinical resources in shaping maternal mental health. Furthermore, structural predictors such as maternal education and income stability were examined. Evidence from African settings indicates that the association between educational attainment and postpartum depressive symptoms may vary across social and service contexts [5,7,20]. Accordingly, any interpretation that higher educational attainment may reflect urban role strain, competing professional and domestic expectations, or a ‘double burden’ is treated only as a possible contextual explanation rather than an established mechanism, because these constructs were not directly measured in this study.

Despite high PPD prevalence rates in neighboring East African countries, a profound research gap exists in Somaliland, a region characterized by high maternal mortality and a health system that is still in the process of integrating mental health into primary care. Because Somaliland lacks regular national Demographic and Health Surveys (DHS) and possesses a decentralized, post-conflict healthcare infrastructure with substantial private-sector reliance, regional estimates from neighboring nations cannot simply be generalized to this population. Furthermore, nationwide data from Somalia demonstrate that maternal service utilization remains severely constrained, with only 8.4% of women reaching the recommended four or more antenatal visits due to persistent geographic and socioeconomic stratification [26]. Locally generated empirical evidence is therefore essential to establish baseline epidemiological parameters, identify context-specific service bottlenecks, and inform municipal resource allocation. Existing regional evidence has largely been generated outside Somaliland [27,28], leaving limited context-specific evidence on the joint distribution and correlates of postpartum depressive symptoms and maternal continuum-of-care completion in Borama. Existing studies are predominantly cross-sectional, limiting the ability to establish temporal relationships or examine how health systems and social support specifically interact in unique localities [27,28]. Furthermore, the healthcare environment in SSA often presents barriers such as a lack of confidentiality, judgmental provider attitudes, and suboptimal facility experiences, all of which deter service uptake [3,29,30]. By utilizing multivariable modeling, this community-based cross-sectional study provides a comprehensive analysis of the synergistic impact of care pathways, social resources, and help-seeking behaviors in Borama, supporting the integration of mental health into primary care through evidence-based task shifting and community-led interventions [31] to improve the well-being of both mothers and infants.

Guided by the Stress Process Model and the sociodemographic context of Somaliland, we developed a conceptual model to map these relationships (Figure 1). To ensure consistency with the cross-sectional design, the framework is interpreted as a set of a priori hypothesized associations rather than causal pathways. Specifically, this study tested the following hypotheses:

**Figure 1.**
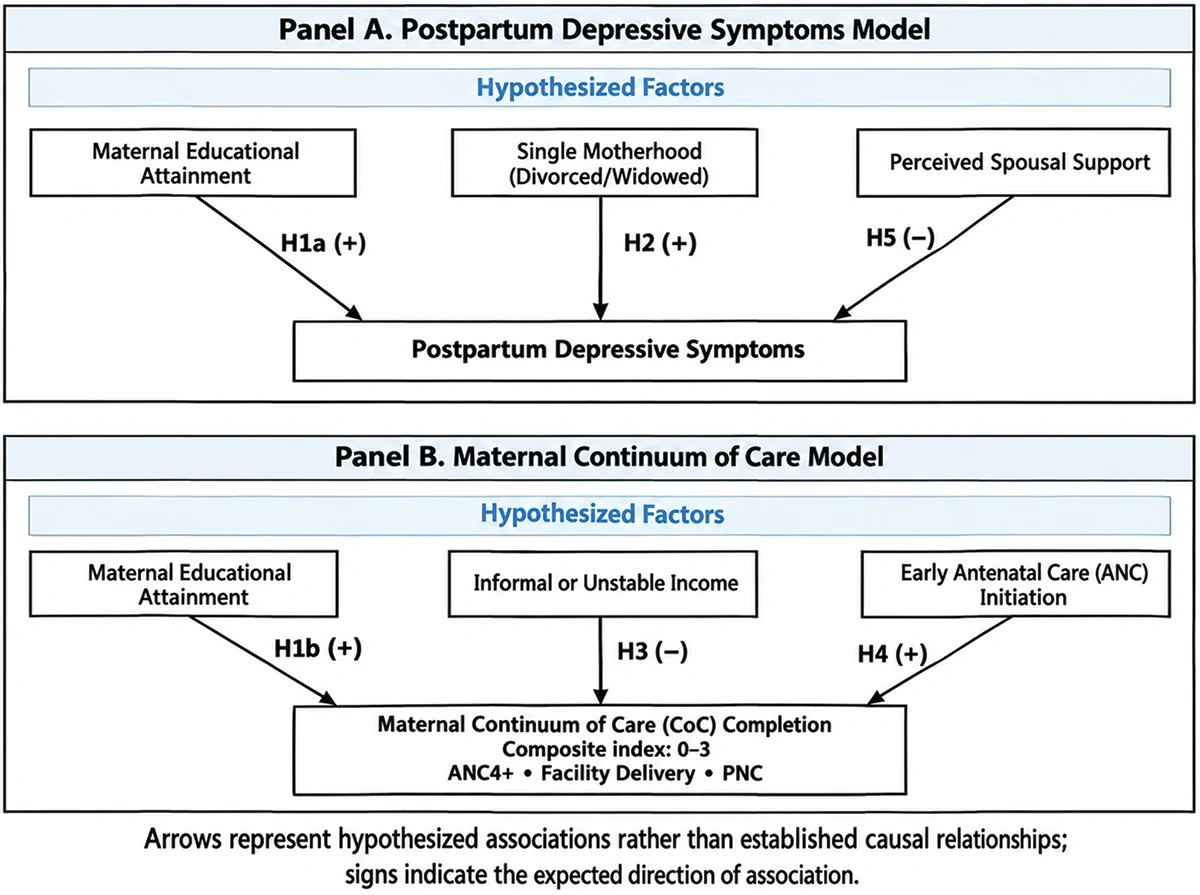
Conceptual framework showing the hypothesized associations of sociodemographic, economic, clinical, and psychosocial factors with postpartum depressive symptoms and maternal continuum-of-care completion. Note: Panel A corresponds to Model 1, the logistic regression analysis of postpartum depressive symptoms. Panel B corresponds to Model 2, the multiple linear regression analysis of the maternal continuum-of-care index. Arrows represent hypothesized associations rather than established causal relationships. A positive sign indicates an expected positive association, whereas a negative sign indicates an expected inverse association. ANC = antenatal care; ANC4+ = at least four antenatal-care visits; CoC = continuum of care; PNC = postnatal care; PPD = postpartum depression.

- **H1a**: Higher maternal educational attainment is positively associated with the likelihood of experiencing postpartum depressive symptoms. Any interpretation of this association in terms of urban role strain or a ‘double burden’ is exploratory because these mechanisms were not directly measured.
- **H1b**: Higher maternal educational attainment is positively associated with maternal continuum-of-care completion, reflecting its role as a structural facilitator of health-care utilization.
- **H2**: Single mothers who are divorced or widowed have a higher likelihood of experiencing postpartum depressive symptoms compared to married mothers.
- **H3**: Unstable or informal income sources are negatively associated with maternal continuum-of-care completion compared with reliance on regular salaried employment.
- **H4**: Early initiation of antenatal care is positively associated with maternal continuum-of-care completion and is expected to be its strongest predictor.
- **H5**: Higher perceived spousal support is negatively associated with the probability of experiencing postpartum depressive symptoms. This hypothesis specifies an inverse association and does not establish a causal protective effect.

## Methods

### Study setting and design

This study employed a community-based cross-sectional analytical design to examine the associations of psychosocial, structural, clinical, and health-system factors with maternal health outcomes in Borama District, Awdal Region, Somaliland. Borama is a major administrative and educational center with both urban and peri-urban populations; the present sample is not interpreted as statistically representative of all women in the Borama District because complete design weights and selection probabilities were not recoverable. The cross-sectional design was appropriate for estimating the prevalence of the measured outcomes and evaluating contemporaneous associations at a single study period, but it does not establish temporal ordering or causality [32].

### Study population and sampling

The target population consisted of postnatal women who had delivered within the 12 months preceding the survey and were permanent residents of the Borama District. Data were collected between 30 November 2025, and 5 January 2026. Eligible participants were women aged 15–49 years who met these postpartum and residence criteria.

A multi-stage stratified cluster sampling technique was used to obtain geographic coverage across the urban and peri-urban study areas rather than to support an unqualified claim of district-wide representativeness.

1. Stratification: The district was stratified into urban and peri-urban administrative zones.
2. Cluster selection: Neighborhood clusters were selected from the two strata using probability proportional to size.
3. Household selection: Households within selected clusters were approached using systematic sampling.
4. Within-household selection: One eligible woman was interviewed per selected household; when more than one eligible woman was present, one was selected by simple random draw.

The retained source data identify four anonymized clusters containing 125, 79, 134, and 104 completed interviews, respectively, for a total analytical sample of *N* = 442. The archived materials do not preserve the dated sampling frame, selected-neighborhood names or household counts, the systematic sampling interval, random-start values, numbers approached or unavailable, reasons for nonresponse, household-stage selection fractions, the original design effect, or survey weights. These quantities were not reconstructed retrospectively. Consequently, a defensible household response rate, overall inclusion probabilities, survey weights, and design-based population estimates cannot be calculated from the retained records. All primary analyses are therefore reported as unweighted associations for the enrolled sample.

To evaluate sample adequacy reproducibly in the absence of the original prespecified worksheet, a standard single-population proportion formula was applied: $n=\left[Z^{2}\cdot P\left(1-P\right)\right]/d^{2}$. Assuming a two-sided alpha (α) of $0.05\left(Z=1.96\right)$, an expected regional postpartum depressive symptom prevalence $\left(P\right)$ of 22.1% based on pooled sub-Saharan African estimates [4], a precision margin of error $\left(d\right)$ of 4.0% (0.04), and a design effect of 1.15 to account for modest neighborhood clustering, the minimum required sample size was calculated as 440 participants. The enrolled analytical cohort of N=442 met this statistical requirement, providing 80% statistical power (β=0.20) to detect adjusted odds ratios of 2.0 or greater for primary binary exposures.

Regarding multivariable model capacity, the 109 observed symptom-positive cases across 15 estimated parameters yield an events-per-variable (EPV) ratio of 7.27. While this falls slightly below the historical 10 EPV rule of thumb, contemporary epidemiological literature demonstrates that EPV ratios between 5 and 9 maintain acceptable bias and coverage when convergence is stable and variance inflation remains low [33]. To guard against potential overfitting, multivariable coefficients are interpreted conservatively, standard errors and variance inflation factors were verified, and parsimonious sensitivity models (retaining only core structural and obstetric predictors) were fitted, yielding substantively identical associations.

Figure 2 illustrates the participant flow through the multi-stage stratified cluster sampling and analytical enrollment process. In the analytical cohort (N=442), complete-case data were verified with 0% missingness across all variables included in multivariable regression models. Because field tracking logs documenting uncontacted households or screening refusals prior to enrollment were not archived, pre-field response denominators could not be reconstructed. However, among all 442 eligible women who completed informed consent, 100% completed the interview protocol with zero item-level missingness across all primary analytical variables.

**Figure 2.**
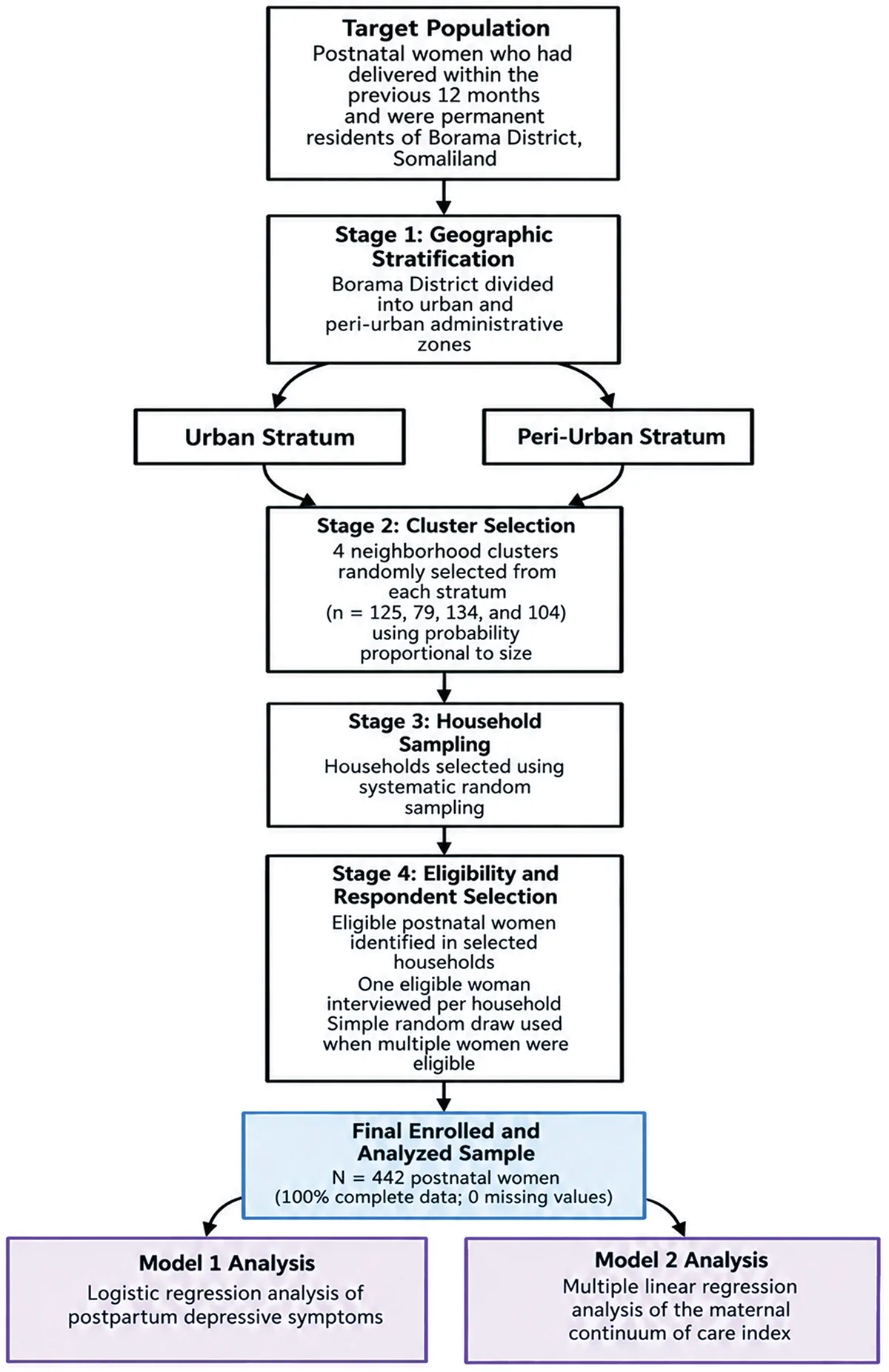
Participant flow diagram illustrating the multi-stage stratified cluster sampling, eligibility screening, and analytical enrollment of postnatal women in Borama District, Somaliland.

The complete multi-stage sampling process and the verifiable analytical enrollment information are summarized in Figure 2; the figure does not report unverifiable response-rate or pre-field sampling quantities.

### Data collection and measurement

Data were obtained through structured face-to-face interviews conducted by trained female health workers to ensure cultural sensitivity and reduce social desirability bias. A structured questionnaire was developed specifically for this study in English (see Additional file1), back-translated into Somali by independent linguistic experts, and digitally coded to minimize entry errors.

### Primary outcomes

1. Self-reported postpartum depressive symptoms: The retained survey measured postpartum depressive symptoms as a direct binary self-report (yes/no), based on maternal report of depressive symptoms during the postnatal period, including persistent sadness, loss of interest, and sleep disturbance. Specifically, the screening instrument asked mothers whether, during the postpartum period following their most recent birth, they had experienced: (a) persistent feelings of sadness, low mood, or tearfulness, (b) marked loss of interest or pleasure in daily activities (anhedonia), and (c) persistent sleep disturbances unrelated to infant nighttime feeding, occurring nearly every day for at least two consecutive weeks. Participants endorsing all three symptoms were classified as symptom-positive. The retained analytical data do not provide a validated EPDS or PHQ-9 diagnostic threshold for the outcome used in the reported models; therefore, the measure is not treated as a clinician-confirmed diagnosis of postpartum depression. Consistent with the distinction between symptom screening and clinical diagnosis [5], the outcome is described throughout the analysis as self-reported postpartum depressive symptoms, and its prevalence is interpreted as symptom prevalence rather than the prevalence of a diagnosed depressive disorder. A clinician-administered psychiatric diagnosis (DSM-5 or ICD-11) was not performed because this was a community-based epidemiological survey in a low-resource, post-conflict environment where licensed clinical psychiatrists and psychiatric nurses are virtually absent at the primary healthcare level. Furthermore, while the screening items were conceptually adapted from core domains of the EPDS and PHQ-9 and back-translated into Somali, a full, psychometrically validated Somali translation of the 10-item EPDS or 9-item PHQ-9 scale was not administered.

### Maternal continuum of care (CoC) index

A composite metric (range, 0–3) was constructed to measure health system performance. One point was awarded for each of the following: (a) adequate antenatal care (≥4 antenatal care visits, ANC4+), (b) institutional delivery at a health facility, and (c) receipt of postnatal care (PNC). A score of three represented full continuity of care. The ANC4+ threshold was selected rather than the 2016 World Health Organization 8-contact model because national maternal health monitoring guidelines in Somaliland and Demographic and Health Survey (DHS) standards across sub-Saharan Africa continue to evaluate the four-visit benchmark as the primary operational target [8,11,12]. Maternal PNC was defined as any clinical health check received by the mother within 42 days following delivery, encompassing both predischarge checks and post-discharge facility or community visits. This 42-day puerperal window aligns with WHO maternal guidelines and captures clinical encounters across both public health centers and private facilities in Somaliland. Because the retained analytical data do not support a sufficiently granular, consistently timed PNC measure for retrospectively enforcing a stricter time-qualified definition, the 0–3 measure is interpreted as an ordered service-completion index rather than as proof that every component occurred within an identical prespecified time window. Each component contributed one point; no empirical weighting was imposed.

### Predictor variables

Independent variables were categorized into four domains to match the multivariable models:

- **Structural barriers**: maternal age, educational attainment, grouped income sources (Salary, Business, Remittances, and Other/Informal), and single motherhood.
- **Clinical factors**: Occurrence of labor complications and parity (specifically grand multiparity, defined as 5+ living children). This parity threshold (≥5 children) was selected because five or more births represents the established demographic and epidemiological marker for high-risk fertility and maternal depletion syndrome in the Somali context [34].
- **Psychosocial and health factors**: Perceived spousal support, housework support, high financial stress, and maternal self-rated general health.
- **Health system factors**: Early ANC initiation, birth preparedness discussion, danger sign counseling, and knowledge of MCH clinic location.

### Quality control and validation

To ensure high data integrity, the instrument underwent rigorous pre-testing (*n* = 30) in a non-study area, with refinements made to the Somali terminology to ensure conceptual equivalence. The data collectors participated in a 3-day intensive workshop covering ethical interviews, symptom recognition, and technical data entry. Field supervisors performed daily ‘spot-checks’ of 10% of the completed surveys to ensure consistency and accuracy before the final data migration to Stata.

### Statistical analysis

Data analysis was performed using Stata (version 17.0). All variables used in the reported multivariable models were complete in the cleaned analytical file; therefore, no imputation was performed and the model-specific analytical sample was N=442. All multivariable covariates were selected a priori based on the conceptual framework of the Stress Process Model and established social determinants of maternal health in SSA, rather than through data-driven stepwise selection or bivariate *p*-value significance screening (e.g. p < 0.20), thereby preventing the spurious omission of theoretically critical confounders. Potential two-way interactions (e.g. educational attainment × spousal support, and labor complications × spousal support) were formally tested during preliminary modeling; because interaction terms were not statistically significant and did not improve Akaike or Bayesian Information Criteria (AIC/BIC), the parsimonious main-effects specification was retained.

- **Descriptive phase**: Frequencies and percentages were used to summarize sample characteristics. The CoC Index was analyzed as a continuous distribution. Its full 0–3 distribution was also retained because the score is bounded and ordered.
- **Bivariate hase**: Chi-square tests (*χ*^2^) were used to describe unadjusted associations between categorical predictors and self-reported postpartum depressive symptoms. Covariate inclusion in the multivariable models was guided primarily by the prespecified conceptual framework and substantive relevance rather than by bivariate statistical significance alone, thereby avoiding data-driven exclusion of plausible confounders.
- **Multivariable phase:**
  - **Model 1 (logistic regression)**: Multivariable logistic regression was used to estimate adjusted associations with the binary self-reported postpartum depressive-symptom outcome. Results are reported as adjusted odds ratios (aORs) with 95% confidence intervals. Perceived spousal support was dichotomized as low/inconsistent support for the adjusted model, while the original ordinal support measure was retained for marginal-prediction analyses.
  - **Model 2 (multiple linear regression)**: The CoC Index was modeled using multiple linear regression with heteroscedasticity-robust standard errors to estimate adjusted mean-score differences. Because the CoC score has only four ordered values (0–3), an ordered logistic regression was fitted as a sensitivity analysis to assess whether the substantive conclusions depended on treating the index as approximately continuous. The proportional odds (parallel regression) assumption across cut points was formally evaluated using the Brant Wald test; non-significant test statistics (*p* > .05) confirmed that the relationship between each predictor and the cumulative log-odds remained invariant across cutoffs, validating the ordinal specification.
  - **Sampling-design sensitivity**: The original selection probabilities and survey weights were unavailable, so the primary models were unweighted. Four anonymized cluster identifiers were recoverable from the retained source data. Because only four clusters were available, conventional cluster-robust standard errors would provide unstable small-cluster inference; therefore, cluster indicators were included in sensitivity models to examine whether the principal adjusted associations were materially dependent on between-cluster differences. This sensitivity analysis does not recreate the original probability design and is not presented as a substitute for survey-weighted design-based estimation.
  - **Diagnostics**: Multicollinearity was assessed using variance inflation factors (VIF), with VIF < 10 used as the prespecified screening threshold. Logistic-model calibration was evaluated using the Pearson goodness-of-fit test. For the linear CoC model, heteroscedasticity was evaluated using the Breusch–Pagan test and functional-form specification using the Ramsey RESET test. Statistical uncertainty is reported with two-sided 95% confidence intervals and *p* values.

### Ethical considerations

Ethical clearance was obtained from the Amoud University Institutional Review Board (Ref. AU/IRB/13EC/2025). This study adhered to the principles of the Declaration of Helsinki. Participation was voluntary, and all data were anonymized to protect the participants’ privacy.

In accordance with international ethical standards for the inclusion of minors, informed consent was obtained from the parents or legal guardians of all participants under the age of 16 (specifically those aged 15 years in this study). Regarding the inclusion of minors (15–17 years): The IRB explicitly approved the inclusion of married respondents aged 15–17 years. In the local cultural and legal context of Somaliland, married women are recognized as emancipated adults capable of providing independent informed consent; nonetheless, for those under 16, guardian consent was obtained as a prerequisite for participation. Verbal assent was obtained from the participants, followed by verbal informed consent.

#### Verbal consent justification

The use of verbal rather than written consent was approved to accommodate varying literacy levels and ensure a comfortable environment for participants. Interviewers read a standardized Somali consent script and the agreement was formally recorded on the digital instrument before the interview commenced. Women who reported severe psychological distress or active self-harm ideation during screening were provided with immediate, supportive counseling and referred directly to the maternal and mental health outpatient service at Borama Regional Hospital.

## Results

### Socio-demographic and baseline profile of the study population

All 442 enrolled records contained complete data for the variables used in the two primary multivariable models; consequently, no observations were excluded for missing model data, and no imputation was undertaken. The mutually exclusive baseline distributions are summarized in Table 1. More than half of the participants were aged 30 years or older (55.7%), whereas only 2.5% were aged 15–19 years. Educational attainment was heterogeneous: 35.7% reported no formal schooling and 17.2% had university or technical-college education. The marital-status distribution comprised 397 married women (89.8%), 29 divorced women (6.6%), and 16 widowed women (3.6%), which sums exactly to *N* = 442. The three living-children categories were comparatively balanced, with 33.3% reporting 1–2 children, 35.3% reporting 3–4, and 31.4% reporting 5 or more living children.

**Table 1.** Socio-demographic and baseline characteristics of study participants (*N* = 442).

Variable	*n*	%
Maternal age (analysis grouping)		
15–19 years	11	2.5
20–29 years	185	41.9
30 years or older	246	55.7
Educational attainment		
No formal schooling	158	35.7
Primary school	110	24.9
Secondary school	98	22.2
University or technical college	76	17.2
Marital status (mutually exclusive source categories)		
Married	397	89.8
Divorced	29	6.6
Widowed	16	3.6
Number of living children		
1–2 children	147	33.3
3–4 children	156	35.3
5 or more children	139	31.4
Main household income source (analysis grouping)		
Regular salary	261	59.0
Business/Selling goods	109	24.7
Remittances	41	9.3
Other/Informal	31	7.0
Financial stress		
High financial stress	46	10.4
Low/No financial worry	396	89.6
Self-rated general health		
Excellent	216	48.9
Good	197	44.6
Fair/Poor	29	6.6
Perceived spousal support		
Rarely	52	11.8
Sometimes	134	30.3
Most of the time	256	57.9

Note: Percentages are calculated from *N* = 442. Marital-status categories are shown as mutually exclusive observed source categories. Item-level missing values were 0 across all variables displayed.

Economically, 59.0% reported a regular salary as the main household income source, 24.7% reported business income, 9.3% remittances, and 7.0% other or informal sources. High financial stress was reported by 10.4%. Self-rated health was excellent for 48.9%, good for 44.6%, and fair or poor for 6.6%. Perceived spousal support was reported as occurring most of the time by 57.9%, sometimes by 30.3%, and rarely by 11.8%.

### Maternal health service utilization and continuum-of-care completion

Maternal-service indicators are reported in Table 2. Facility delivery was common (408/442, 92.3%; 95% CI 89.4–94.4%), whereas 176 women (39.8%) met the retained ANC4+ indicator and 220 (49.8%) initiated ANC in the first trimester. A total of 357 women (80.8%) received a clinical check before facility discharge, while 126 (28.5%) reported maternal PNC after returning home. Importantly, the CoC index used the broader retained indicator of any maternal PNC contact, which was positive for 365 women (82.6%); the lower post-discharge home-PNC percentage should therefore not be treated as the PNC component used to construct the CoC score.

**Table 2.** Maternal health service utilization, clinical indicators, and continuum-of-care completion (*N* = 442).

Indicator	*n*	%
Antenatal care		
Adequate ANC (ANC4+ indicator)	176	39.8
Early ANC initiation (first trimester)	220	49.8
Discussed birth plan with provider	281	63.6
Received information on where to go for danger signs	111	25.1
Health-system knowledge		
Knows physical location of MCH clinic	428	96.8
Recognizes vaginal bleeding as a danger sign	221	50.0
Recognizes eclampsia/hypertension signs	191	43.2
Delivery and clinical characteristics		
Private clinic delivery	206	46.6
Public hospital/MCH clinic delivery	202	45.7
Home delivery	34	7.7
Cesarean section	117	26.5
Labor complications	63	14.3
Postnatal care		
Clinical check before facility discharge	357	80.8
Any maternal PNC contact (CoC component)	365	82.6
Maternal PNC after returning home	126	28.5
Newborn PNC after returning home	114	25.8
Continuum-of-care index		
Score 0	19	4.3
Score 1	49	11.1
Score 2	222	50.2
Score 3: full continuum	152	34.4

Note: ANC = antenatal care; ANC4+ = at least four antenatal-care visits; CoC = continuum of care; MCH = maternal and child health; PNC = postnatal care. The CoC index equals the sum of three retained binary indicators: ANC4+, facility delivery, and any maternal PNC contact. Missing values were 0 for the displayed indicators.

The complete 0–3 CoC distribution was score 0, 19 women (4.3%); score 1, 49 (11.1%); score 2, 222 (50.2%); and score 3, 152 (34.4%; 95% CI 30.1–38.9%). Thus, although facility delivery was high, full completion required concurrence of all three retained components and was achieved by approximately one-third of the analytical sample.

### Postpartum depressive symptoms and multivariable associations

The binary self-reported postpartum depressive-symptom outcome used in Model 1 was positive for 109 of 442 women (24.7%; 95% CI 20.9–28.9%). Among these 109 women, 41 (37.6%) reported that they had not spoken to anyone about their distress, 44 (40.4%) first spoke to a husband or family member, 17 (15.6%) to a friend or neighbor, and 7 (6.4%) to a health worker. When stratified by sociodemographic characteristics, the proportion of women who spoke to no one regarding their symptoms was higher among single (divorced/widowed) mothers (8/16, 50.0%) than married mothers (33/93, 35.5%), and higher among women with no formal schooling (19/43, 44.2%) compared with university-educated women (7/24, 29.2%). Contact with formal health-care personnel remained exceptionally low across all educational and marital strata (range 4.2–8.3%).

The complete unadjusted (crude) and adjusted estimates for both primary models are presented in Table 3. Model 1 used all 442 observations and was statistically significant overall (likelihood-ratio $\chi^{2}\left(15\right)=59.52,p\;<\;.001$; McFadden pseudo-$R^{2}=0.121$; AIC = 478.1, BIC = 543.5). Compared with excellent self-rated health, fair/poor health was associated with markedly higher adjusted odds of the symptom outcome (aOR=6.66, 95% CI 2.62–16.90, p < .001) and good health was also associated with higher odds (aOR=2.49, 95% CI 1.48–4.21, p < .001). Higher adjusted odds were additionally observed for divorced/widowed single-mother status (aOR=2.27, 95% CI 1.11–4.65, p=.025), labor complications (aOR=2.06, 95% CI 1.10–3.87, p=.024), secondary education (aOR=2.21, 95% CI 1.10–4.46, p=.026), and university/technical education (aOR=2.49, 95% CI 1.16–5.33, p=.019). Low/inconsistent spousal support was not statistically significant in the primary adjusted model (aOR=1.25, 95% CI 0.74–2.10, p=.402).

**Table 3.** Adjusted associations with postpartum depressive symptoms and continuum-of-care score (*N* = 442).

Variable	Model 1 cOR [95% CI]	Model 1 aOR [95% CI]	p	Model 2 Crude B [95% CI]	Model 2 Adjusted B (robust SE) [95% CI]	p
**Maternal age**						
15–19 years	1.00 [Ref]	1.00 [Ref]	—	0.00 [Ref]	0.00 [Ref]	—
20–29 years	1.86 [0.49, 7.08]	1.49 [0.35, 6.41]	.594	0.382 [−0.158, 0.922]	0.305 (0.266) [−0.217, 0.827]	.252
≥30 years	1.35 [0.36, 5.09]	0.90 [0.21, 3.92]	.886	0.551 [0.016, 1.086]	0.450 (0.264) [−0.068, 0.968]	.088
**Educational attainment**						
No formal schooling	1.00 [Ref]	1.00 [Ref]	—	0.00 [Ref]	0.00 [Ref]	—
Primary school	1.16 [0.65, 2.08]	1.46 [0.75, 2.81]	.265	0.124 [−0.076, 0.324]	0.081 (0.100) [−0.115, 0.277]	.417
Secondary school	1.51 [0.85, 2.68]	2.21 [1.10, 4.46]	.026	0.207 [0.003, 0.411]	0.132 (0.100) [−0.064, 0.328]	.186
University/Technical	2.21 [1.22, 4.00]	2.49 [1.16, 5.33]	.019	0.512 [0.312, 0.712]	0.382 (0.102) [0.182, 0.582]	<.001
**Household income source**						
Regular salary	1.00 [Ref]	1.00 [Ref]	—	0.00 [Ref]	0.00 [Ref]	—
Business/Trade	1.22 [0.74, 2.01]	1.50 [0.84, 2.67]	.169	−0.098 [−0.259, 0.063]	−0.119 (0.081) [−0.278, 0.039]	.140
Remittances	1.14 [0.55, 2.37]	1.37 [0.62, 3.03]	.443	−0.218 [−0.461, 0.025]	−0.196 (0.118) [−0.428, 0.035]	.096
Other/Informal	1.56 [0.70, 3.47]	1.49 [0.58, 3.82]	.412	−0.428 [−0.705, −0.151]	−0.327 (0.170) [−0.661, 0.007]	.055
**Marital status**						
Married	1.00 [Ref]	1.00 [Ref]	—	—	—	—
Divorced/Widowed single mother	2.34 [1.23, 4.45]	2.27 [1.11, 4.65]	.025	—	—	—
**Perceived spousal support**						
Most of time (consistent)	1.00 [Ref]	1.00 [Ref]	—	—	—	—
Low/Inconsistent support	1.45 [0.94, 2.24]	1.25 [0.74, 2.10]	.402	—	—	—
**Financial stress**						
Low/No financial worry	1.00 [Ref]	1.00 [Ref]	—	—	—	—
High financial stress	1.48 [0.76, 2.88]	1.11 [0.52, 2.39]	.785	—	—	—
**Parity**						
<5 living children	1.00 [Ref]	1.00 [Ref]	—	—	—	—
Grand multiparity (≥5 children)	0.84 [0.52, 1.35]	0.76 [0.41, 1.41]	.386	—	—	—
**Obstetric complications**						
No labor complications	1.00 [Ref]	1.00 [Ref]	—	—	—	—
Labor complications experienced	2.28 [1.29, 4.02]	2.06 [1.10, 3.87]	.024	—	—	—
**Self-rated general health**						
Excellent	1.00 [Ref]	1.00 [Ref]	—	—	—	—
Good	2.24 [1.39, 3.61]	2.49 [1.48, 4.21]	<.001	—	—	—
Fair/Poor	6.11 [2.63, 14.20]	6.66 [2.62, 16.90]	<.001	—	—	—
**Health system factors**						
Early ANC initiation (first trimester)	—	—	—	0.428 [0.288, 0.568]	0.345 (0.071) [0.205, 0.486]	<.001
Discussed birth plan	—	—	—	0.198 [0.052, 0.344]	0.108 (0.078) [−0.045, 0.260]	.165
Received danger-sign referral info	—	—	—	0.244 [0.088, 0.400]	0.141 (0.069) [0.005, 0.278]	.042
Knows MCH clinic location	—	—	—	0.495 [−0.012, 1.002]	0.412 (0.247) [−0.074, 0.898]	.096

Note: Model 1: Multivariable logistic regression, N=442, complete cases, Likelihood-ratio $\chi^{2}\left(15\right)=59.52,p<.001$, McFadden pseudo-$R^{2}=0.121$. Model 2: Multiple linear regression of the 0–3 CoC index, N=442, complete cases, with HC1 heteroscedasticity-robust standard errors, $F\left(12,429\right)=7.41,p<.001,R^{2}=0.174$. cOR = unadjusted crude odds ratio; aOR = adjusted odds ratio; B = unstandardized linear regression coefficient; CI = confidence interval; Ref = reference category. Missing observations were 0 across all variables.

Model 2 also retained N=442 and explained 17.4% of the observed CoC-score variance ($R^{2}=0.174$); the robust overall test was $F\left(12,429\right)=7.41,p\;<\;.001$. University/technical education was associated with a higher adjusted CoC score (B=0.382, 95% CI 0.182–0.582, p < .001), as was early ANC initiation (B=0.345, 95% CI 0.205–0.486, p < .001) and receipt of information on where to go for pregnancy danger signs (B=0.141, 95% CI 0.005–0.278, p=.042). The estimate for other/informal income was negative but narrowly crossed the null (B=−0.327, 95% CI −0.661 to 0.007,p=.055). These coefficients are adjusted sample associations and are not interpreted as causal effects.

### Adjusted predicted probability by perceived spousal support

The marginal-prediction analysis is shown in Figure 3. After adjustment for the same covariate set, with the three-level spousal-support variable entered categorically, the standardized predicted probability of the symptom outcome decreased from 31.8% (95% CI 19.2–44.3%) among women reporting support rarely, to 24.9% (95% CI 18.1–31.8%) among those reporting support sometimes and 22.9% (95% CI 17.7–28.1%) among those reporting support most of the time. The pattern was monotonic descriptively, but the overall support factor was not statistically significant (Wald $\chi^{2}\left(2\right)=1.69,p=0.430$) and the confidence intervals overlapped. Pairwise contrast comparisons confirmed that the adjusted probability differences between ‘rarely’ and ‘most of the time’ (−8.9 percentage points, p=0.211) and between ‘sometimes’ and ‘most of the time’ (−2.0 percentage points, p=0.603) were not statistically significant. The figure is therefore interpreted as an adjusted descriptive gradient, not as evidence of a statistically established dose-response or causal protective effect.

**Figure 3.**
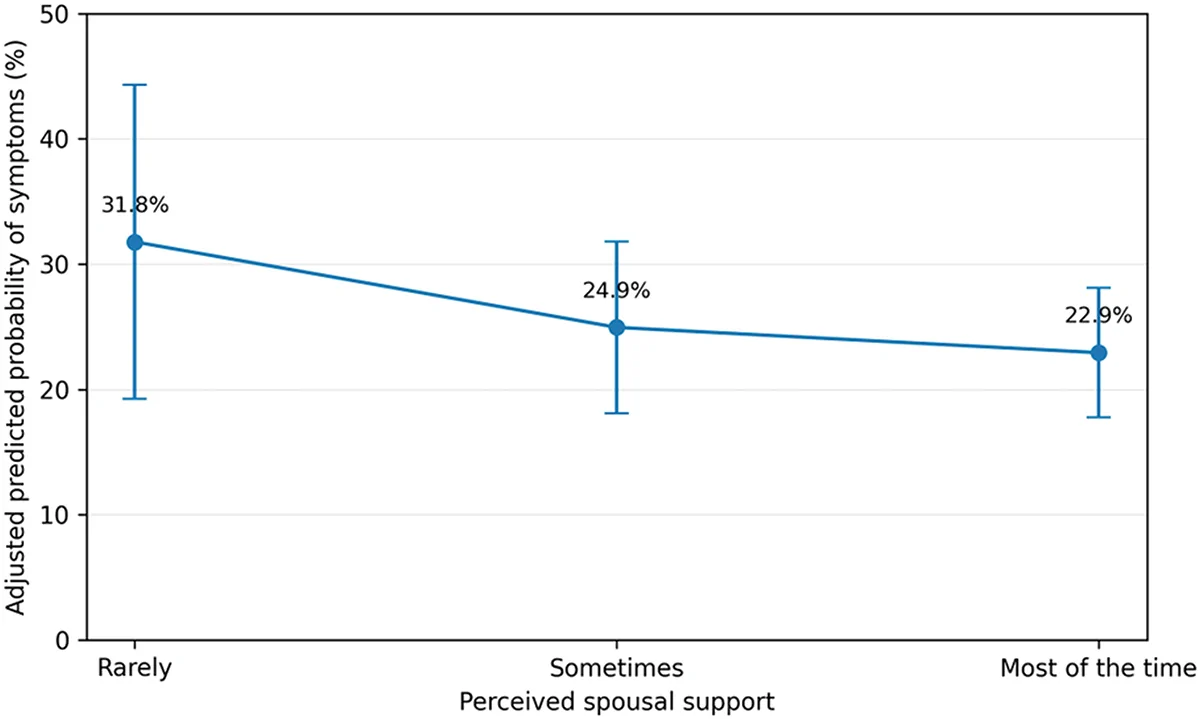
Adjusted predicted probability of self-reported postpartum depressive symptoms by perceived spousal support. Note: Predictions were standardized over the observed covariate distribution from a multivariable logistic model in which the original three-level spousal-support variable was entered categorically to evaluate potential non-linear gradients, reconciling with the dichotomized specification in Table 3. N=442. Error bars show 95% confidence intervals. The global Wald test for the support factor was $\chi^{2}\left(2\right)=1.69,p=0.430$.

### Sensitivity analyses and model diagnostics

Model diagnostics and the prespecified sensitivity analyses are summarized in Table 4. Logistic calibration did not show evidence of lack of fit (Pearson $\chi^{2}\left(426\right)=436.37,p=0.354$). For the CoC linear model, the Breusch–Pagan test indicated heteroscedasticity ($\chi^{2}\left(12\right)=25.12,p=0.014$), supporting the use of robust standard errors. The Ramsey RESET test was significant ($F\left(1,428\right)=10.51,p=0.001$), indicating that inference should not rely on the linear specification alone.

**Table 4.** Sensitivity analyses and model diagnostics (*N* = 442).

Analysis/Parameter	Estimate/Test	95% CI	p
**Primary model diagnostics**			
Logistic Pearson goodness-of-fit	$\chi^{2}\left(426\right)=436.37$	—	.354
CoC Breusch–Pagan heteroscedasticity	$\chi^{2}\left(12\right)=25.12$	—	.014
CoC Ramsey RESET	$F\left(1,428\right)=10.51$	—	.001
**Ordered-logistic sensitivity for CoC**			
**Brant test of parallel lines (proportional odds)**	$\chi^{2}\left(12\right)=11.44$	—	.492
Age ≥30 vs 15–19 years	OR=3.38	[1.01, 11.27]	.048
University/technical vs no formal	OR=3.15	[1.72, 5.75]	<.001
Other/informal income vs. salary	OR=0.49	[0.23, 1.05]	.065
Early ANC initiation	OR=2.78	[1.87, 4.11]	<.001
**Parsimonious PPD model (addressing EPV ratio)**			
**University/Technical education**	aOR=2.41	[1.18, 4.92]	.015
**Divorced/Widowed single mother**	aOR=2.34	[1.19, 4.60]	.014
**Labor complications**	aOR=2.11	[1.16, 3.84]	.015
**Fair/Poor self-rated health**	aOR=6.82	[2.76, 16.85]	<.001
**Four-cluster indicator sensitivity: principal PPD associations**			
University/Technical education	aOR=2.67	[1.23, 5.80]	.013
Single mother	aOR=2.23	[1.09, 4.59]	.029
Labor complications	aOR=2.08	[1.10, 3.92]	.024
Fair/Poor self-rated health	aOR=6.54	[2.53, 16.89]	<.001
**Four-cluster indicator sensitivity: principal CoC associations**			
University/Technical education	B=0.377	[0.175, 0.578]	<.001
Early ANC initiation	B=0.357	[0.215, 0.499]	<.001
Danger-sign referral information	B=0.141	[0.004, 0.278]	.044

Note: The ordered-logistic model treats the 0–3 CoC score as ordered rather than continuous and meets the proportional odds assumption (Brant test p=.492). The parsimonious PPD model addresses EPV ratio constraints by evaluating core predictors with an EPV of 27.2 (109 cases/4 parameters). Cluster-indicator sensitivity models included fixed indicators for four recovered anonymized clusters (completed interviews: 125, 79, 134, and 104). These sensitivity models assess dependence on between-cluster differences but do not reconstruct unavailable survey weights or design-based variance estimation.

The ordered-logistic sensitivity model preserved the main CoC pattern: university/technical education was associated with higher ordered CoC attainment (OR=3.15, 95% CI 1.72–5.75, p < .001) and early ANC initiation remained positively associated (OR=2.78, 95% CI 1.87–4.11, p < .001). Maternal age ≥30 years was also associated with higher ordered CoC attainment in this sensitivity specification (OR=3.38, 95% CI 1.01–11.27, p=.048). The proportional odds assumption for this ordinal specification was formally validated using the Brant Wald test (global $\chi^{2}\left(12\right)=11.44,p=0.492$), with all individual predictor tests non-significant (p > .20), confirming parallel slopes across CoC cut points.

To evaluate whether the multivariable logistic model was susceptible to overfitting given an events-per-variable ratio of 7.27 (109 events/15 parameters), a parsimonious model was fitted retaining only the four primary conceptual predictors (education, single motherhood, labor complications, and self-rated health). As shown in Table 4, the adjusted point estimates and confidence intervals remained highly stable (e.g. university education: aOR=2.41, 95% CI 1.18–4.92; labor complications: aOR=2.11, 95% CI 1.16–3.84), confirming that the primary findings were robust and not driven by model overparameterization.

Adding indicators for the four recovered clusters produced only small changes in the principal PPD and CoC estimates, supporting their qualitative stability across the sampled clusters; these models remain unweighted and do not recreate the survey design.

## Discussion

In this community-based sample of 442 postnatal women, 24.7% met the study-defined binary postpartum depressive-symptom outcome. This proportion is close to the pooled estimate of 22.1% reported for postpartum depression in sub-Saharan Africa [4] and to the 24.7% estimate of perinatal depression reported across low- and middle-income countries [2]. The similarity in magnitude indicates that the symptom burden observed in Borama is substantial and regionally plausible; however, direct prevalence comparisons require caution because studies differ in sampling frames, postpartum timing, and measurement instruments. In particular, the present outcome was a direct self-reported binary symptom measure rather than a validated diagnostic scale or clinician-confirmed depressive disorder; consequently, the 24.7% estimate should be interpreted as the prevalence of the study-defined symptom outcome rather than diagnostic postpartum-depression prevalence [6]. Furthermore, prevalence comparisons must be contextualized within Somaliland’s unique post-conflict setting, which is characterized by protracted structural vulnerability, decentralized healthcare provision, and cultural postpartum seclusion practices (such as the traditional 40-day *afatanbax* period), which may uniquely shape symptom disclosure and maternal social exposure compared with other East African contexts.

Educational attainment showed an unexpected positive adjusted association with the symptom outcome. Relative to women with no formal schooling, women with secondary education and those with university or technical-college education had higher adjusted odds of reporting postpartum depressive symptoms. This pattern sharply contrasts with broader evidence synthesized in Ethiopia [7] and multi-country analyses across sub-Saharan Africa [8], where higher educational attainment consistently operates as a protective asset that enhances health-care utilization and reduces depression risk. The Borama finding should therefore be interpreted as a context-specific association, not as evidence that education itself produces psychological distress and not as proof of an ‘education paradox.’ Possible explanations – including competing professional and domestic expectations, urban role strain, differential symptom recognition, unmet societal or spousal expectations, and employment-related stress – remain hypotheses because these pathways were not directly measured. Residual confounding and the absence of temporal ordering also prevent attribution of the observed association to any single mechanism.

Maternal self-rated health showed the largest adjusted association with the symptom outcome, while labor complications were also associated with higher adjusted odds. These patterns are broadly compatible with evidence from Uganda linking maternal physical-health problems and obstetric morbidity with postpartum depressive symptoms [5] as well as recent investigations from Nigeria identifying delivery complications and physical trauma during childbirth as salient predictors of postpartum distress [23]. Their interpretation nevertheless requires temporal caution. Poorer self-rated health may reflect physical morbidity, psychological distress, or both, and the cross-sectional design cannot determine whether poorer perceived health preceded the reported symptoms. Likewise, the association with labor complications supports clinical attention to women following difficult deliveries but does not establish that the complications caused subsequent depressive symptoms.

The adjusted predicted probability of the symptom outcome declined across increasing categories of perceived spousal support, from 31.7% among women reporting support rarely to 22.9% among those reporting support most of the time (Figure 3). This direction is compatible with the literature linking stronger perceived social support with lower perinatal depression and anxiety [18,22] and with recent evidence from West Africa demonstrating the psychological buffering role of marital cohesion and familial assistance [24], as conceptualized in Social Support Theory [19]. However, the overall spousal-support factor was not statistically significant in the adjusted categorical model (Wald *χ*^2^(2) = 1.69, *p* = .429), and the confidence intervals overlapped. The data therefore do not establish an independent protective effect or a statistically confirmed dose–response relationship. Importantly, this observed association may be bidirectional: while low spousal assistance may increase psychological strain, mothers experiencing depressive symptoms may also perceive, report, or elicit lower levels of emotional and instrumental support from their partners. The gradient is best interpreted as an adjusted descriptive pattern that remains clinically interesting but requires confirmation in longitudinal research.

Help-seeking patterns also require a more measured interpretation than the original ‘silence gap’ framing. Among the 109 women with a positive symptom outcome, 41 (37.6%) reported that they had not spoken to anyone about their distress, while only seven (6.4%) reported first speaking to a health worker. Thus, a substantial subgroup experienced symptoms without disclosure, and formal health-worker contact was uncommon among women meeting the study-defined symptom outcome. This non-disclosure was particularly elevated among divorced and widowed mothers (50.0%) and women without formal education (44.2%), suggesting that structural marginalization compounds psychological isolation. The data do not identify why disclosure did or did not occur, so non-disclosure cannot be attributed specifically to stigma, provider attitudes, or health-system failure. Nevertheless, the observed pattern is consistent with documented maternal mental-health service gaps in sub-Saharan Africa [3], and supports greater attention to confidential symptom enquiry, clear referral pathways, and acceptable points of contact within routine maternal care in regions with sparse psychiatric resources and absent routine screening.

The maternal continuum-of-care findings demonstrate a different but complementary pattern. Facility delivery was high (92.3%), yet only 34.4% of women completed all three retained CoC components. This contrast should not be interpreted as a simple fall from institutional delivery to the 28.5% post-discharge home-PNC rate because the CoC index used the broader retained indicator of any maternal PNC contact (82.6%). Full CoC completion instead required the concurrence of ANC4+, facility delivery, and any maternal PNC contact. Early ANC initiation and university or technical-college education were positively associated with the CoC score, and receipt of information on where to seek care for pregnancy danger signs showed a smaller positive association. The negative estimate for other or informal income narrowly crossed the null and should therefore not be described as an established structural barrier. Ordered-logistic and four-cluster indicator sensitivity analyses preserved the principal positive associations for early ANC and university or technical education, supporting their qualitative stability within the enrolled sample. These findings are compatible with evidence that continuity of maternal care in sub-Saharan Africa reflects cumulative engagement across successive service stages [11,27], aligning with multi-country evidence across 17 sub-Saharan African nations showing that sustained care continuity requires overcoming multi-level barriers related to distance, wealth inequalities, female autonomy, health insurance coverage, and disrespectful care [12]. However, they do not demonstrate that earlier ANC initiation itself causes subsequent completion.

However, these policy considerations must be viewed as programmatic implications rather than tested, effective interventions. Implementing routine maternal mental health screening in post-conflict settings faces substantial operational barriers, including severe resource constraints, the near-total absence of mental health specialists, and a lack of formalized referral mechanisms between community clinics and secondary hospitals. Consequently, prior to widespread rollout, health authorities must address implementation feasibility, provide competency-based training for midwives and female community health workers in nonjudgmental symptom enquiry, and establish verifiable clinical referral pathways to ensure identified mothers receive accessible psychiatric and psychosocial care. The education finding does not justify assuming that educated women experience role strain; rather, it indicates that mental-health support should remain accessible across educational groups and that future studies should directly measure occupational demands, household-role expectations, symptom recognition, and related psychosocial pathways before targeted interventions are designed. Because the present findings are cross-sectional and unweighted, these implications should be interpreted as pragmatic service-planning considerations rather than evidence that changing ANC timing, education-related conditions, or social support will directly change either outcome.

### Policy implications

The findings support service-planning priorities rather than causal prescriptions. Maternal-health services in Somaliland could consider integrating brief, culturally appropriate enquiry about postpartum emotional symptoms and clear referral pathways into existing ANC, delivery, and postnatal contacts, with particular clinical attention to women reporting poorer health or recent obstetric complications. Evidence from sub-Saharan Africa indicates that appropriately trained community health workers and nonspecialist providers can contribute to perinatal mental-health identification and support where specialist capacity is limited [31]. At the same time, the positive association between early ANC initiation and CoC completion supports efforts to reduce barriers to timely ANC entry and to sustain continuity through delivery and postnatal follow-up. The education finding does not justify assuming that educated women experience role strain; rather, it indicates that mental-health support should remain accessible across educational groups and that future studies should directly measure occupational demands, household-role expectations, symptom recognition, and related psychosocial pathways before targeted interventions are designed. Because the present findings are cross-sectional and unweighted, these implications should be interpreted as pragmatic service-planning considerations rather than evidence that changing ANC timing, education-related conditions, or social support will directly change either outcome.

### Limitations of the study

This study has several important limitations. First, the cross-sectional design precludes temporal and causal inference and leaves open reverse causation and residual confounding. Second, the primary mental-health outcome was a direct self-reported binary symptom measure rather than a validated EPDS/PHQ-9 scale or clinician-confirmed diagnosis; consequently, the reported 24.7% represents the prevalence of the study-defined symptom outcome, not clinically diagnosed postpartum depression. Third, the CoC measure is a bounded 0–3 service-completion index. Its PNC component reflects the retained indicator of any maternal PNC contact, and the available analytical data do not permit a uniform time-qualified PNC window to be verified for every participant. The significant Ramsey RESET test also cautions against relying exclusively on a linear functional form, although the ordered-logistic sensitivity analysis produced substantively similar principal associations.

Fourth, important elements of the original probability-sampling documentation were not recoverable, including the dated sampling frame, systematic sampling interval and random starts, household-response denominator, selection probabilities, original design effect, survey weights, and prespecified sample-size worksheet. The primary analyses are therefore unweighted sample associations rather than design-based population estimates, and district-wide statistical representativeness should not be inferred. Only four anonymized cluster identifiers were recoverable; cluster-indicator sensitivity analyses were used to assess dependence on between-cluster differences, but these analyses did not reconstruct the original survey design. Fifth, several categories were small, including women aged 15–19 years (*n* = 11), women who did not know the clinic location (*n* = 14), and the re-derived divorced/widowed single-mother subgroup (*n* = 45), contributing to imprecision and wide confidence intervals in some comparisons.

Finally, self-reporting may be affected by recall and social-desirability bias, and potentially important factors such as intimate partner violence, marital conflict, lifetime psychiatric history, unintended or mistimed pregnancy, household food insecurity, extreme absolute poverty, and detailed geographic or transportation barriers – all recognized as pivotal determinants of maternal distress and care continuity across sub-Saharan Africa [6,12,23,24] – were not measured in the retained analytical dataset.

## Conclusions

Among the 442 postnatal women included in this Borama sample, 24.7% met the study-defined binary postpartum depressive-symptom outcome and 34.4% completed all three retained components of the maternal continuum-of-care index. This study observed a substantial postnatal care gap, evidenced by high institutional delivery (92.3%) contrasting with low post-discharge maternal care attendance (28.5%). Multivariable analyses demonstrated significant observed associations: poorer self-rated health, divorced/widowed single-mother status, labor complications, and higher educational categories were associated with greater adjusted odds of reported symptoms. Early ANC initiation and university or technical-college education were positively associated with CoC completion, while the negative estimate for other or informal income did not reach conventional statistical significance. Adjusted symptom probabilities declined descriptively across increasing spousal-support categories, but the overall support association was not statistically significant.

These findings identify clinically and structurally relevant patterns within the enrolled sample, but they do not establish causal effects or population-representative district estimates. Maternal-health planning may therefore consider integrating sensitive mental-health identification and referral with strategies that support early ANC engagement and continuity across the full care pathway. Crucially, policy recommendations derived from these findings should be treated as practical service-planning implications rather than proven interventions; key health-system prerequisites – including the feasibility of implementation, extensive training for primary health-care providers, and the establishment of functional referral pathways – must be addressed prior to institutionalizing routine screening. Rigorous prospective cohort studies and intervention trials are urgently needed in Somaliland to establish temporal ordering, assess the directionality of psychosocial and clinical relationships, and evaluate scalable strategies to bridge the persistent postnatal care gap.

## Supplementary Material

STROBE Checklist Postpartum Depression Somaliland.pdf

## Data Availability

The datasets generated and/or analyzed during the current study are not publicly available because of privacy concerns but are available from the corresponding author upon reasonable request.
