# Supplementary material for: Psychosocial and structural factors associated with postpartum depressive symptoms and maternal continuum of care in Borama, Somaliland: a cross-sectional study
Source: Glob Health Action. 2026 Oct 7;19(1):2741766. doi: 10.1080/16549716.2026.2741766 (PMC13647386; doi:10.1080/16549716.2026.2741766)
Supplement: Supplementary file 1 [file ZGHA_A_2741766_SM6552.pdf]

STROBE Statement - checklist of items that should be included in reports of cross-sectional studies

	Item No.	Recommendation	Page No.	Relevant text from manuscript
Title and abstract	1	(a) Indicate the study's design with a commonly used term in the title or the abstract.	1	Title includes "A Cross-Sectional Study in Borama, Somaliland."
		(b) Provide in the abstract an informative and balanced summary of what was done and what was found.	1	The structured abstract reports the design, N=442, principal PPD and CoC results, and conclusion.
**Introduction**				
Background/rationale	2	Explain the scientific background and rationale for the investigation being reported.	2-3	Background covers PPD burden, maternal CoC gaps, social support, and the Somaliland evidence gap.
Objectives	3	State specific objectives, including any prespecified hypotheses.	3-4	The conceptual model is followed by prespecified hypotheses H1a-H5.
**Methods**				
Study design	4	Present key elements of study design early in the paper.	5	The study is identified as a "community-based cross-sectional analytical design."
Setting	5	Describe the setting, locations, and relevant dates, including periods of recruitment, exposure, follow-up, and data collection.	5	Borama District, Awdal Region; data collected from 30 November 2025 to 5 January 2026.
Participants	6(a)	Give the eligibility criteria, and the sources and methods of selection of participants.	5-6	Eligible postnatal women had delivered within the previous 12 months and were permanent residents; multistage household sampling was used.
Variables	7	Clearly define all outcomes, exposures, predictors, potential confounders, and effect modifiers. Give diagnostic criteria, if applicable.	7-8	PPD, the CoC index, four predictor domains, and covariate screening are described; no effect modifier was specified.
Data sources/measurement	8*	For each variable of interest, give sources of data and details of methods of assessment (measurement). Describe comparability of assessment methods if there is more than one group.	6-7	Structured face-to-face questionnaire, Somali translation, outcome definitions, and CoC scoring are described.
Bias	9	Describe any efforts to address potential sources of bias.	5-7, 17	Probability sampling, one respondent per household, female interviewers, pretesting, training, spot checks, and bias limitations are reported.
Study size	10	Explain how the study size was arrived at.	5	N=442 is described as sufficient for more than 15 predictors and stable standard errors; no formal calculation is provided.

Quantitative variables	11	Explain how quantitative variables were handled in the analyses. If applicable, describe which groupings were chosen and why.	7-8, 9-12	CoC was analyzed continuously; support was dichotomized for regression and retained ordinally for marginal effects; category boundaries are shown.
Statistical methods	12(a)	Describe all statistical methods, including those used to control for confounding.	7-8	Stata 17; descriptive statistics, chi-square screening, multivariable logistic and linear regression, robust standard errors, CIs, and diagnostics.
	12(b)	Describe any methods used to examine subgroups and interactions.	N/A	No subgroup or interaction analysis was reported.
	12(c)	Explain how missing data were addressed.	NR	The missing-data procedure is not explicitly described; tables and models state N=442.
	12(d)	If applicable, describe analytical methods taking account of sampling strategy.	5-6; NR	The multistage stratified cluster design is described, but analytical adjustment for clustering or stratification is not stated.
	12(e)	Describe any sensitivity analyses.	8, 14	Ordered logistic regression was used as a sensitivity analysis of the OLS CoC model.
**Results**				
Participants	13(a)*	Report numbers of individuals at each stage of study - for example, potentially eligible, examined for eligibility, confirmed eligible, included, and analysed.	5-6, 9	Final enrolled and analyzed N=442; counts at earlier screening stages are not provided.
	13(b)*	Give reasons for non-participation at each stage.	NR	Reasons for non-participation are not reported.
	13(c)*	Consider use of a flow diagram.	6	Figure 2 presents the participant sampling and analytical flow.
Descriptive data	14(a)*	Give characteristics of study participants (for example, demographic, clinical, and social characteristics) and information on exposures and potential confounders.	9-12	Tables 1-2 report demographic, economic, psychosocial, clinical, and health-service characteristics.
	14(b)*	Indicate the number of participants with missing data for each variable of interest.	NR	Variable-specific missing counts are not reported; Tables 1-2 state N=442.
Outcome data	15*	Report numbers of outcome events or summary measures.	11-13	PPD symptoms: 24.7% (n=109); full CoC: 34.4% (n=152).
Main results	16(a)	Give unadjusted estimates and, if applicable, confounder-adjusted estimates and their precision (for example, 95% confidence intervals). Make clear which confounders were adjusted for and why they were included.	13-15	Adjusted estimates with 95% CIs or standard errors are reported; model covariates are listed. Unadjusted model estimates are not reported.
	16(b)	Report category boundaries when continuous variables were categorized.	7, 9-12	Thresholds/categories for age, parity, CoC, income, financial stress, social resilience, ANC timing, and service indicators are shown.
	16(c)	If relevant, consider translating estimates of relative risk into absolute risk for a meaningful time period.	15	Predicted PPD probability declines from 31.8% to 22.9% across levels of spousal support.

Continued on next page

Other analyses	17	Report other analyses done - for example, analyses of subgroups and interactions, and sensitivity analyses.	14-15	Ordered-logit sensitivity results and the marginal-effects analysis of spousal support are reported.
**Discussion**				
Key results	18	Summarise key results with reference to study objectives.	16-18	The Discussion and Conclusions summarize PPD, CoC, structural barriers, clinical gateways, and psychosocial resilience.
Limitations	19	Discuss limitations of the study, taking into account sources of potential bias or imprecision. Discuss both direction and magnitude of any potential bias.	17	Cross-sectional design, self-report, a small single-mother subgroup, one district, and unmeasured variables are discussed; bias magnitude is not quantified.
Interpretation	20	Give a cautious overall interpretation of results considering objectives, limitations, multiplicity of analyses, results from similar studies, and other relevant evidence.	16-18	Interpretation compares regional evidence, discusses mechanisms and implications, and uses cautious association language.
Generalisability	21	Discuss the generalisability (external validity) of the study results.	17	The authors state that Borama findings may not reflect rural or nomadic populations.
**Other information**				
Funding	22	Give the source of funding and the role of the funders for the present study and, if applicable, for the original study on which the present article is based.	19	The study received no specific grant from public, commercial, or not-for-profit funding agencies.

***** Give information separately for exposed and unexposed groups where applicable. The present study evaluates multiple predictors rather than one defined exposed/unexposed cohort.

Reporting key: NR = not reported; N/A = not applicable. Page numbers refer to the supplied 22-page manuscript PDF.

Note: The STROBE checklist should be used with the Explanation and Elaboration article. Information on the STROBE Initiative is available at www.strobe-statement.org.
